# The value of radiomics-based hyperdense middle cerebral artery sign in predicting hemorrhagic transformation in acute ischemic stroke patients undergoing endovascular treatment

**DOI:** 10.3389/fneur.2024.1492089

**Published:** 2024-12-23

**Authors:** Chundan Gong, Yun Liu, Wei Ma, Yang Jing, Li Liu, Yan Huang, Jinlin Yang, Chen Feng, Yuan Fang, Weidong Fang

**Affiliations:** ^1^Department of Radiology, The First Affiliated Hospital of Chongqing Medical University, Chongqing, China; ^2^Department of Radiology, The People's Hospital of Yubei District of Chongqing City, Chongqing, China; ^3^Medical Imaging Department, Chongqing University Central Hospital, Chongqing Emergency Medical Center, Chongqing, China; ^4^Huiying Medical Technology Co., Ltd., Beijing, China; ^5^Department of Radiology, Chongqing Traditional Chinese Medicine Hospital, Chongqing, China

**Keywords:** radiomics, hyperdense middle cerebral artery sign, hemorrhagic transformation, acute ischemic stroke, endovascular treatment

## Abstract

**Objective:**

To establish and validate a model based on hyperdense middle cerebral artery sign (HMCAS) radiomics features for predicting hemorrhagic transformation (HT) in patients with acute ischemic stroke (AIS) after endovascular treatment (EVT).

**Methods:**

Patients with AIS who presented with HMCAS on non-contrast computed tomography (NCCT) at admission and underwent EVT at three comprehensive hospitals between June 2020 and January 2024 were recruited for this retrospective study. A radiomics model was constructed using the HMCAS radiomics features most strongly associated with HT. In addition, clinical and radiological independent factors associated with HT were identified. Subsequently, a combined model incorporating radiomics features and independent risk factors was developed via multivariate logistic regression and presented as a nomogram. The models were evaluated via receiver operating characteristic curve, calibration curve, and decision curve analysis.

**Results:**

Of the 118 patients, 71 (60.17%) developed HT. The area under the curve (AUC) of the radiomics model was 0.873 (95% CI 0.797–0.935) in the training cohort and 0.851 (95%CI 0.721–0.942) in the test cohort. The Alberta Stroke Program Early CT score (ASPECTS) was the only independent predictor among 24 clinical and 4 radiological variables. The combined model further improved the predictive performance, with an AUC of 0.911 (95%CI 0.850–0.960) in the training cohort and 0.877 (95%CI 0.753–0.960) in the test cohort. Decision curve analysis demonstrated that the combined model had greater clinical utility for predicting HT.

**Conclusion:**

HMCAS-based radiomics is expected to be a reliable tool for predicting HT risk stratification in AIS patients after EVT.

## Introduction

Endovascular thrombectomy (EVT) has been widely used as standard treatment for all eligible patients with acute ischemic stroke (AIS) due to large vessel occlusion (1, 2). Hemorrhage transformation (HT) is the most common and severe complication after EVT, potentially leading lead to a worse prognosis than the natural course of the disease, thus negating the benefits of surgery (3, 4). The European Cooperative Acute Stroke Study II (ECASS II) classifies HT into hemorrhagic infarction (HI) and parenchymal hemorrhage (PH) by imaging (5). The Heidelberg criteria classifies HT into asymptomatic intracranial hemorrhage (ICH) and symptomatic ICH (sICH) according to clinical deterioration (6). However, even minor HT may lead to a poor functional prognosis later in life once HT occurs (7, 8).

Non-contrast computed tomography (NCCT) is the first-line imaging modality for AIS patients because of its rapidity and universality (2). Early signs of intracranial large-vessel occlusive infarction can be detected on admission NCCT, including loss of gray–white matter differentiation at the insula, basal ganglia, and caudate head; loss of the sulcus; and hyperdense middle cerebral artery sign (HMCAS) (9). HMCAS represents acute thromboembolic occlusion of the large vessels [middle cerebral artery (MCA) and/or internal carotid artery (ICA) terminals] with a specificity approaching 100% (10, 11). Some studies have shown that HMCAS is associated with an increased risk of HT after EVT (12, 13). Therefore, it is crucial to identify the risk of hemorrhage in patients presenting with HMCAS prior to EVT, which may guide clinicians in carefully selecting the appropriate treatment regimen and optimizing management to improve patient prognosis.

Radiomics can tap into a wide range of quantitative imaging features in biomedical images that are not recognized by the human eye for use in clinical diagnosis, decision support and prediction of outcomes (14). Most previous radiomics studies on the prediction of HT after reperfusion were based on the brain parenchyma on CT or MRI (15–17). Unclear boundaries of hyperacute-phase cerebral infarcts on NCCT at admission led to uncertainty in the outlining of the region of interest (ROI), and the time-consuming nature of the MRI examination may hinder its application in urgent clinical situations. In recent years, thrombus-based radiomics has shown great potential in the estimation of infarct onset time, recanalization after ischemia-perfusion, and assessment of prognosis (18–22). However, the utility of HMCAS-based radiomics for post-EVT HT has not been reported.

In this study, for the first time, we explored HMCAS-based radiomics on NCCT to predict HT in AIS patients who underwent EVT.

## Materials and methods

### Patients

**Figure 1:**
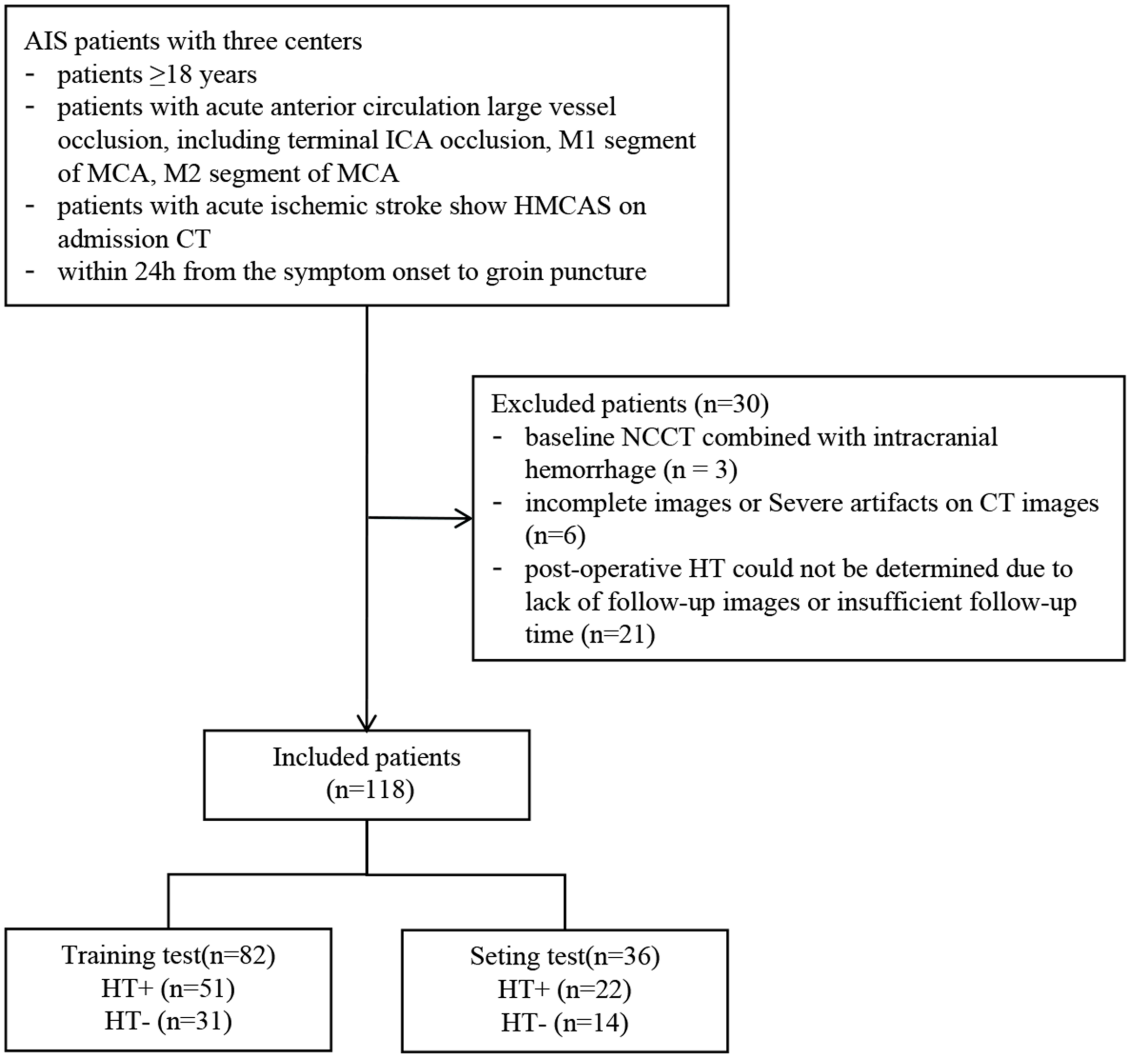
The flow chart of patient recruitment process.

**Figure 2:**
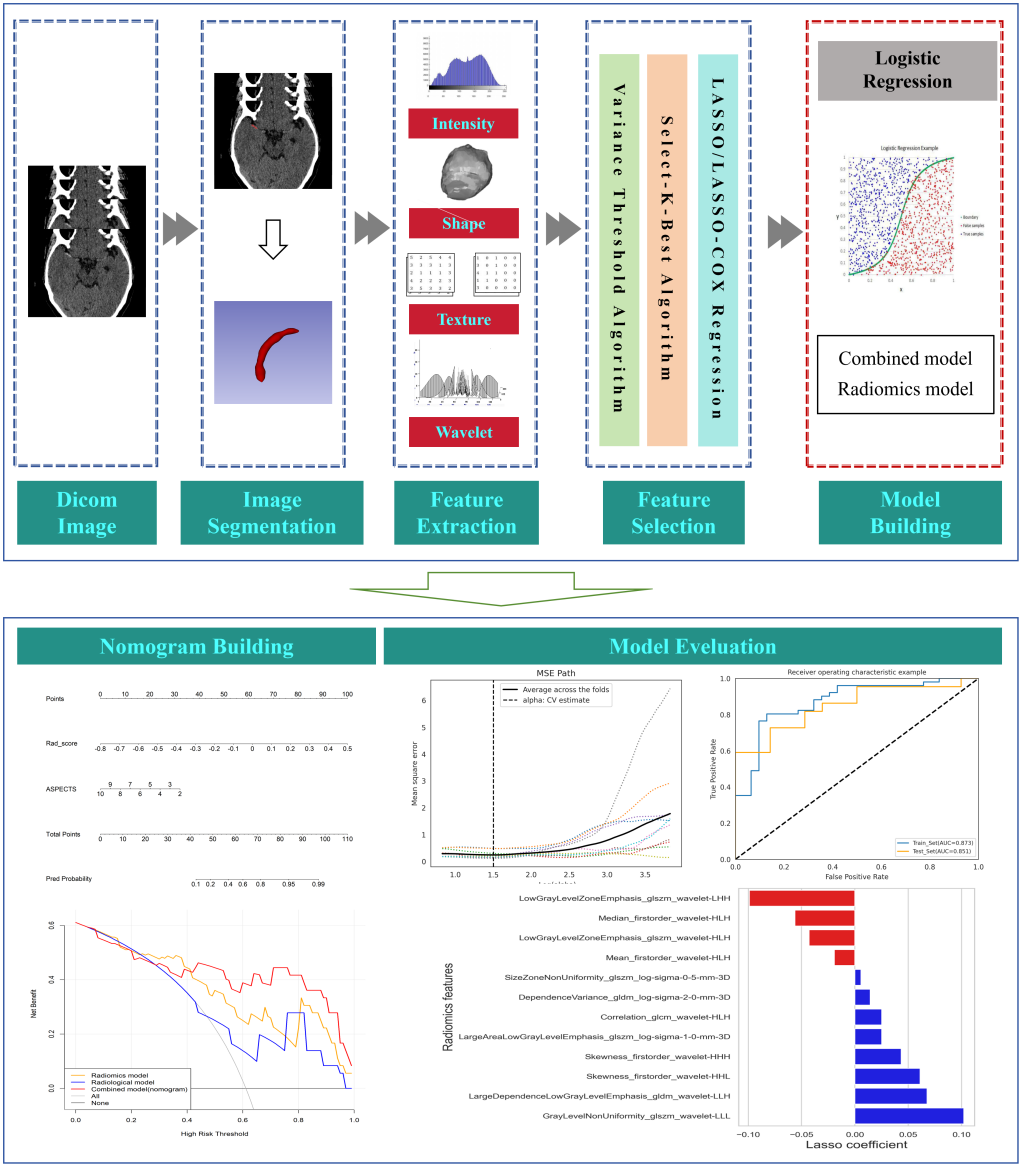
The flow chart of this study.

Imaging and clinical data of AIS patients who underwent EVT in three comprehensive hospitals from June 2020 to January 2024 were retrospectively collected. The inclusion criteria were as follows: (1) age ≥ 18 years; (2) acute anterior circulation large vessel occlusion, including terminal internal carotid artery occlusion, the M1 segment of the MCA, the M2 segment of the MCA; (3) patients with AIS show HMCAS on admission NCCT; and (4) within 24 h from symptom onset to groin puncture. The exclusion criteria were as follows: (1) baseline NCCT combined with intracranial hemorrhage (*n* = 3), (2) severe artifacts on NCCT (*n* = 6), and (3) postoperative HT could not be determined because of a lack of follow-up images or insufficient follow-up time (*n* = 21). Finally, 118 patients were included in this study. All the patients were randomly divided into the training (*n* = 82) and test (*n* = 36) cohorts at a ratio of 7:3. The flow chart of this study is shown in Figures 1, 2.

### Imaging acquisition

The CT scanner models and scanning parameters used in the three comprehensive hospitals are shown in Supplementary Table S1.

### Clinical data collection and imaging evaluation

The following clinical data were collected from all the patients: (1) baseline characteristics, including age, sex, hypertension, diabetes, atrial fibrillation, coronary artery disease, hyperlipidemia, previous stroke, history of anticoagulant drugs, history of antiplatelet drugs, history of smoking, and history of drinking. In addition, the severity of stroke at admission was assessed via the National Institutes of Health Stroke Scale (NIHSS) score, and stroke etiology was determined according to the Trial of Org 10172 in Acute Stroke Treatment (TOAST) (23). (2) Laboratory findings, including triglyceride, cholesterol, high-density lipoprotein (HDL), low-density lipoprotein (LDL), and glucose levels at admission were determined. (3) EVT procedures, including intravenous thrombolysis prior to EVT, thrombectomy mode, angioplasty, number of device passes (>3), and successful recanalization [mTICI 2B and 3 (24)], were also collected and analyzed.

HT was assessed by follow-up CT or MR images within 24 h after EVT. If hyperdense areas, which could not be identified as hemorrhage, were observed on the follow-up NCCT within 24 h, HT was defined as a hyperdense lesion persisting for ≥48 h. If the hyperdense area disappeared or nearly disappeared within 48 h, contrast extravasation was considered (25). We evaluated the following thrombus imaging features: the clot burden score (CBS) (26), the length of the HMCAS, and the distance from the end of the ICA to the thrombus (DT) (27). The CBS is a scoring system used to quantify the thrombosis burden in the anterior circulation on a scale of 0–10; the lower the score is, the more extensive the thrombus. Other imaging data evaluated included the Alberta Stroke Program Early CT Score (ASPECTS) on NCCT, which quantifies early ischemic changes due to middle cerebral artery stroke prior to treatment (28).

### Thrombus segmentation and radiomics feature extraction

The HMCAS was defined if the unilateral middle cerebral artery appeared more dense than the surrounding brain tissue and other arteries but non-calcified on NCCT (29, 30). The region of interest (ROI) of the HMCAS was manually segmented layer-by-layer from axial slices of the NCCT images by a radiologist using 3D Slicer (version 5.6.2) (31). For doubtful areas, the corresponding digital subtraction angiography (DSA) or computed tomography angiography (CTA) images were viewed for guidance, similar to Qiu et al. (19) and Li et al. (21). Prior to feature extraction, the ROIs of all the images were normalized to address the potential effects of inconsistent spatial resolution, including resampling the image voxels to 1 mm × 1 mm × 1 mm by linear interpolation and fixing the bin-width value of the image gray value at 25. Subsequently, 1,130 radiomic features (RFs), including shape features, first-order features, 2D-shaped features, gray-level co-occurrence matrix (GLCM), gray-level dependence matrix (GLDM), gray-level run length matrix (GLRLM), gray-level size zone matrix (GLSZM), and neighboring gray-tone difference matrix (NGTDM), were automatically extracted from all the ROIs via the PyRadiomics plug-in in the software. Wavelet filtering and three different Laplacian Gaussian filters were applied to obtain further higher-order features. Forty patients were randomly selected for the segmentation task to assess the inter-observer agreement of the computationally extracted RFs, and features with an intergroup correlation coefficient (ICC) of less than 0.9 were excluded.

### Feature selection and radiomics model building

Before feature selection, all RFs were normalized, i.e., the mean was removed and divided by its standard deviation, and each set of feature values was converted into normalized data with zero mean and one variance. Owing to the large number of RFs, to avoid problems such as model overfitting and multicollinearity, the extracted RFs were downscaled via the following three methods: first, the variance thresholding method was used for feature dimensionality reduction, and features with variance less than 0.8 were deleted; second, the univariate selection method was utilized to screen for nonsignificant features (*p* > 0.05); and last, through the least absolute shrinkage and selection operator (LASSO) regression algorithm, the indicators most relevant to the research objectives were fitted, and the weights of these indicators were obtained. The radiomic score (Rad score) was calculated via a linear combination of the final sifted features weighted by their respective coefficients, and then a radiomic model was established.

### Development of the radiological and combined models

Clinical and imaging features were screened for variables independently associated with HT via univariate and multivariate logistic regression analyses. Independent risk factors were included in the radiological and combined models. The combined model was visualized by building a radiomic nomogram, which presents the variables included in the model as a column-line graph to provide personalized HT probability estimates.

### Statistical analysis

The Kruskal–Wallis rank sum test or one-way analysis of variance was used for continuous variables. Continuous data are reported as medians [interquartile ranges (IQRs)] or means (standard deviations) depending on whether the data were normally distributed. The chi-square test was used for categorical variables, which are reported as counts (percentages). The receiver operating characteristic (ROC) curve was used to determine the predictive performance of the models. The Delong test was used to compare differences in areas under the curves (AUCs) between the models. Calibration curves and the Hosmer–Lemeshow test were used to assess the calibration performance of the model. The clinical utility of the models was assessed via decision curve analysis (DCA). Statistical analyses were performed via R software (version 4.3.2). A value of *p* < 0.05 was considered to indicate a statistically significant difference.

## Results

### Clinical and radiological characteristics

A total of 118 patients who underwent EVT were included. Among them, 71 (60.17%) patients developed HT (35 [29.66%] with HI and 36 [30.51%] with PH), and 15 (12.71%) patients had sICH. There were no significant differences in any of the variables between the training and test cohorts (*p* > 0.05), indicating an even distribution of data between the two groups (Table 1). In the training set, univariate logistic regression analysis for HT showed that CBS, DT, length of HMCAS, ASPECTS, NIHSS, glucose level, diabetes were significantly difference (*p* < 0.05). However, Only the imaging feature ASPECTS (OR 0.672, 95% CI 0.443–0.946, *p* = 0.039) was an independent predictor for HT in the multivariate logistic regression analysis (Supplementary Table S2). Furthermore, there were no statistically significant differences between patients with HT and without HT in other factors, such as age, gender, number of devices, and successful recanalization (*p* > 0.05).

**Table 1:** Demographics and characteristics of the training and test cohorts.

Variable	Training cohort (*N* = 82)	Test cohort (*N* = 36)	*p* value
Male sex (%)	38 (46.3)	19 (52.8)	0.657
Age (median [IQR])	72.00 [66.50, 82.00]	71.50 [61.75, 82.00]	0.425
Stroke etiologies (%)			0.787
Large artery atherosclerosis	24 (29.3)	12 (33.3)	
Cardioembolism	53 (64.6)	21 (58.3)	
Other etiologies	5 (6.1)	3 (8.3)	
CBS (median [IQR])	7.00 [6.00, 9.00]	6.00[4.75, 8.00]	0.093
DT (median [IQR])	10.35 [0.00, 19.88]	4.00[0.00, 13.75]	0.070
Length of HMCAS (median [IQR])	16.65[10.25, 22.90]	17.60[10.60, 25.25]	0.638
ASPECTS (median [IQR])	9.00 [8.00, 10.00]	9.00[6.00, 10.00]	0.840
Drink (%)	25 (30.5)	17 (47.2)	0.124
Smoke (%)	25 (30.5)	16 (44.4)	0.209
Previous stroke (%)	8 (9.8)	5 (13.9)	0.733
Diabetes (%)	17 (20.7)	8 (22.2)	0.792
Hypertension (%)	46 (56.1)	17 (47.2)	0.491
Atrial fibrillation (%)	59 (72.0)	19 (52.8)	0.070
Coronary heart disease (%)	24 (29.3)	9 (25.0)	0.800
Hyperlipidemia (%)	9 (11.0)	9 (25.0)	0.094
History of anticoagulant drugs (%)	14 (17.1)	3 (8.3)	0.337
History of antiplatelet drugs (%)	12 (14.6)	4 (11.1)	0.824
Baseline NIHSS score [mean (SD)]	15.63 (5.92)	15.06 (6.01)	0.628
Glucose level (median [IQR])	7.18 [5.81, 9.84]	6.84 [5.70, 10.44]	0.891
Triglyceride (median [IQR])	1.08 [0.78, 1.58]	1.10 [0.77, 2.20]	0.291
Cholesterol (median [IQR])	3.86 [3.28, 4.72]	4.20 [3.82, 5.28]	0.026
HDL (median [IQR])	1.08 [0.97, 1.43]	1.15 [1.03, 1.26]	0.498
LDL (median [IQR])	2.21 [1.65, 3.00]	2.58 [2.22, 3.21]	0.094
Intravenous thrombolysis (%)	32 (39.0)	19 (52.8)	0.235
Thrombectomy modes (%)			0.573
Aspiration alone	45 (54.9)	17 (47.2)	
Stent retriever alone	2 (2.4)	2 (5.6)	
Combination of stent retriever and aspiration	35 (42.7)	17 (47.2)	
Angioplasty procedure with stent placement (%)	5 (6.1)	1 (2.8)	0.764
Number of device > 3 (%)	10 (12.2)	3 (8.3)	0.766
Successful recanalization (%)	75 (91.5)	34 (94.4)	0.853

CBS, clot burden score; DT, distance from the end of the ICA to the thrombus; HMCAS, hyperdense middle cerebral artery sign; ASPECTS, the Alberta Stroke Program Early CT Score; HDL, high-density lipoprotein; LDL, low-density lipoprotein.

### Establishment and performance of the radiomics model

**Figure 3:**
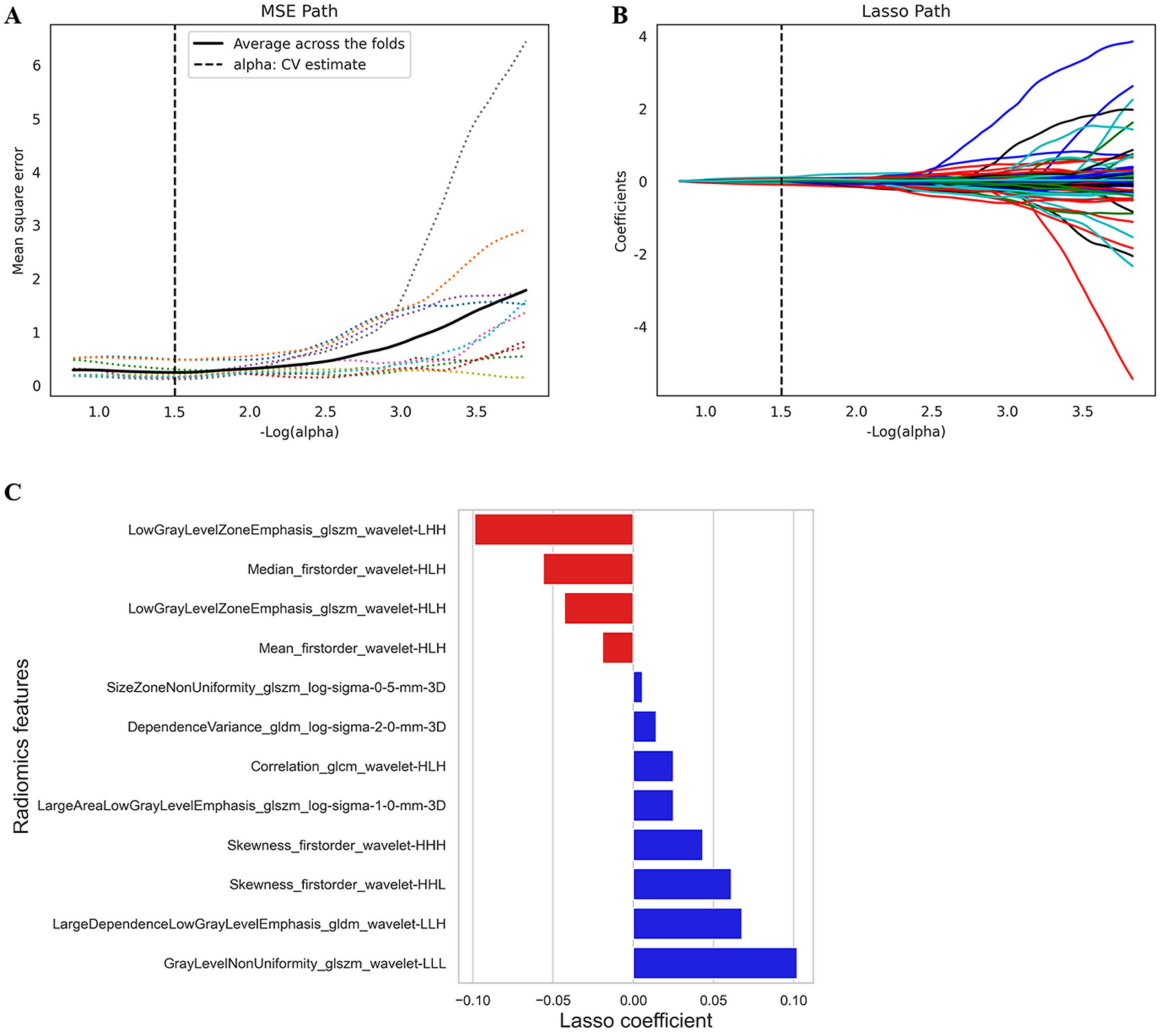
The feature selection by LASSO analysis. **(A)** MSE PATH. The dotted line represents the α value with the smallest mean square error (α = 1.5). **(B)** LASSO PATH. The RFs are determined according to the α value with the smallest mean square error. **(C)** Selected RFs and their corresponding coefficients. MSE, mean squared error; LASSO, least absolute shrinkage and selection operator; RFs, radiomics features.

One thousand one hundred and thirty RFs were extracted from each patient’s ROI, and 205 features with ICCs <0.9 were removed. The 925 features left behind were further reduced by variance thresholding and univariate feature selection methods. Finally, 12 features highly correlated with HT were selected for the radiomics model by LASSO and 10-fold cross-validation (Figures 3A,B). The Rad-score was calculated for each patient via the following formula: Rad-score = 0.622 + (−0.019) × Mean_firstorder_wavelet-HLH + 0.102 × GrayLevelNonUniformity_glszm_wavelet-LLL + (−0.056) × Median_firstorder_wavelet-HLH + (−0.099) × LowGrayLevelZoneEmphasis_glszm_wavelet-LHH + (−0.043) × LowGrayLevelZoneEmphasis_glszm_wavelet-HLH + 0.014 × dependenceVariance_gldm_log-sigma-2-0-mm-3D + 0.061× skewness_firstorder_wavelet-HHL + 0.025 × Correlation_glcm_wavelet-HLH + 0.068 × LargeDependenceLowGrayLevelEmphasis_gldm_wavelet-LLH + 0.025 × LargeAreaLowGrayLevelEmphasis_glszm_log-sigma-1-0-mm-3D +0.006 × SizeZoneNonUniformity_glszm_log-sigma-0-5-mm-3D + 0.043 × Skewness_firstorder_wavelet-HHH (Figure 3C).

**Figure 4:**
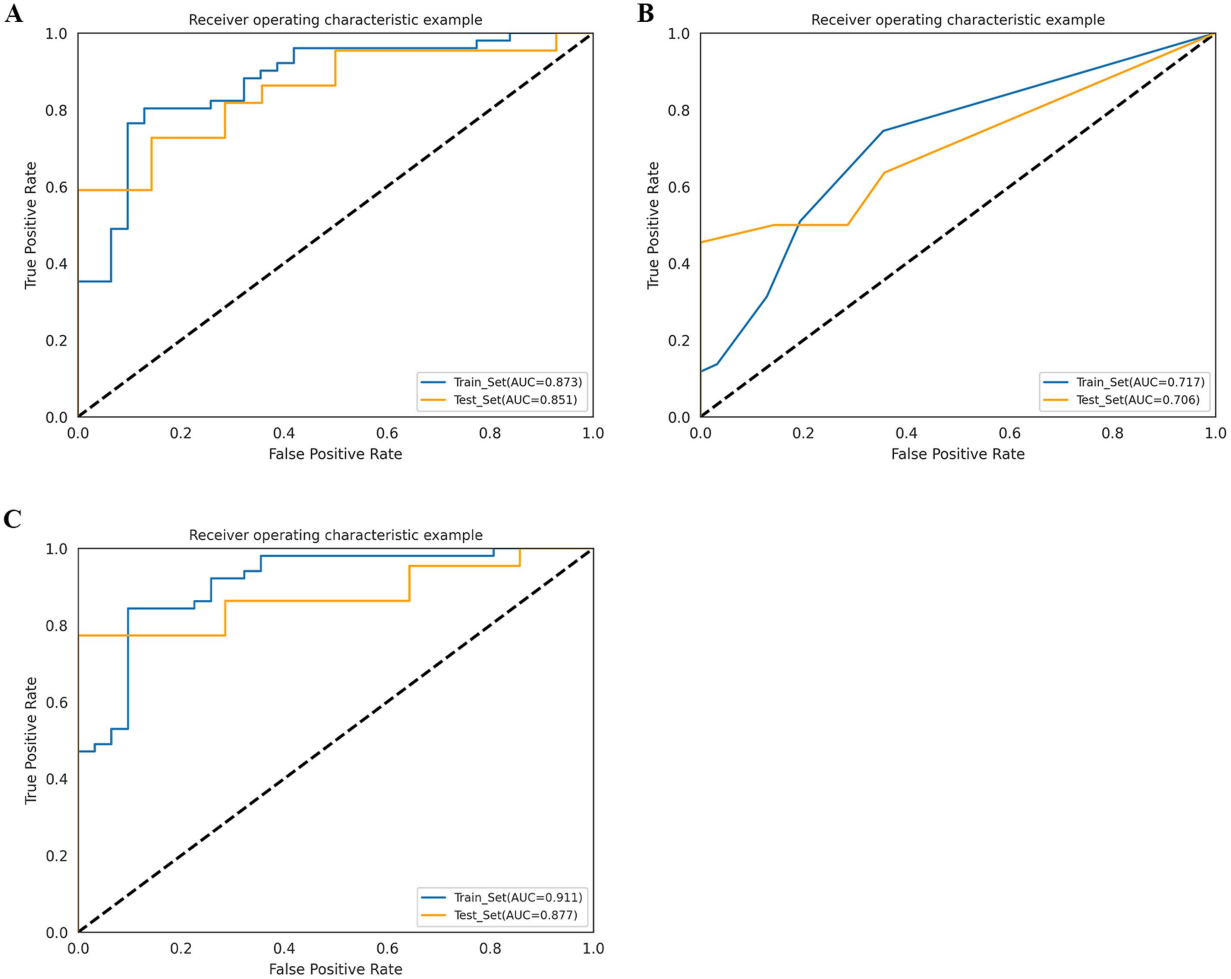
Receiver operating characteristic (ROC) curves of radiomics model **(A)**, radiological model **(B)**, and combined model **(C)** in the training cohort and test cohort.

The AUC of the radiomics model was 0.873 (95% CI: 0.797–0.935) in the training cohort, with an accuracy, sensitivity, and specificity of 0.829, 0.804, and 0.871, respectively. In the test cohort, the AUC was 0.851 (95% CI: 0.721–0.942), with an accuracy, sensitivity, and specificity of 0.722, 0.636, and 0.857, respectively (Table 2; Figure 4).

**Table 2:** Performance of the three models.

Models		AUC (95% CI)	Accuracy	Sensitivity	Specificity
Radiomics model	Training cohort	0.873 (0.797, 0.935)	0.829	0.804	0.871
	Test cohort	0.851 (0.721, 0.942)	0.722	0.636	0.857
Radiological model	Training cohort	0.717 (0.619, 0.805)	0.707	0.745	0.645
	Test cohort	0.706 (0.574, 0.847)	0.639	0.636	0.643
Combined model	Training cohort	0.911 (0.850, 0.960)	0.866	0.843	0.903
	Test cohort	0.877 (0.753, 0.960)	0.778	0.773	0.786

### Establishment and performance of the radiological model

Among the 24 clinical and 4 imaging variables, only the ASPECTS was independently associated with HT, and this imaging variable was included in the radiological model. The AUC of the radiological model was 0.717 (95% CI: 0.619–0.805) in the training cohort and 0.706 (95% CI: 0.574–0.847) in the test cohort (Table 2; Figure 4).

### Establishment and performance of the combined model

We developed a combined model containing an imaging feature (ASPECTS) and the Rad score. The AUC of the combined model was 0.911 (95% CI: 0.850–0.960) in the training cohort, with an accuracy, sensitivity, and specificity of 0.866, 0.843, and 0.903, respectively. In the test cohort, the AUC was 0.877 (95% CI: 0.753–0.960), with an accuracy, sensitivity, and specificity of 0.778, 0.773, and 0.786, respectively (Table 2; Figure 4). The *p* value of the DeLong test for both the combined model and the radiological model was less than 0.05, indicating that the predictive efficiency of the combined model was significantly greater than that of the radiological model in both the training and test cohorts. Although there was no significant difference in predictive efficiency between the combined model and the radiomic model, the AUC value of the combined model was greater than that of the radiomic model (Supplementary Table S3).

**Figure 5:**
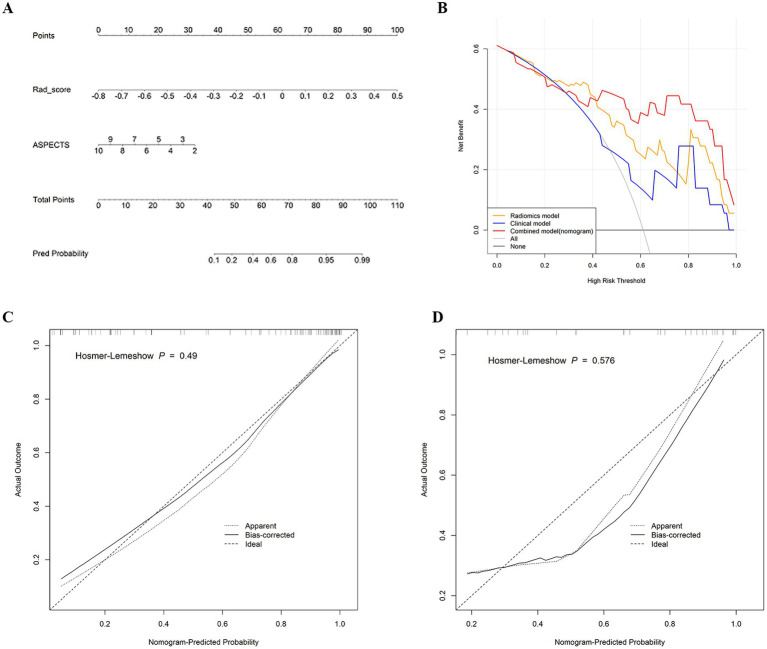
**(A)** Nomogram developed for predicting HT probability based on the combined model, combining two variables: the ASPECTS and Rad-score. **(B)** Decision curve analysis of the three models in the test cohort. y-axis represents the net benefit; x-axis represents the threshold probability. The red, yellow, and blue lines represent the net benefit of the combined model, the radiomics model, and the radiological model, respectively. **(C)** Calibration curve of the nomogram in the training cohort. **(D)** Calibration curve of the nomogram in the test cohort. The dashed line is the reference line for the column plots.

A nomogram was used for visual assessment of the combined model for predicting postoperative HT risk in AIS patients (Figure 5A). Compared with the radiological risk factor, the Rad-score played a major role in the predictive model. Calibration curves and Hosmer–Lemeshow tests revealed that the nomogram had good accuracy in both the training cohort (*p* = 0.49) and the test cohort (*p* = 0.576) (Figures 5C,D). The DCA curve indicated that using the combined model to predict postoperative HT had a greater overall net benefit over large threshold probability interval (Figure 5B).

**Figure 6:**
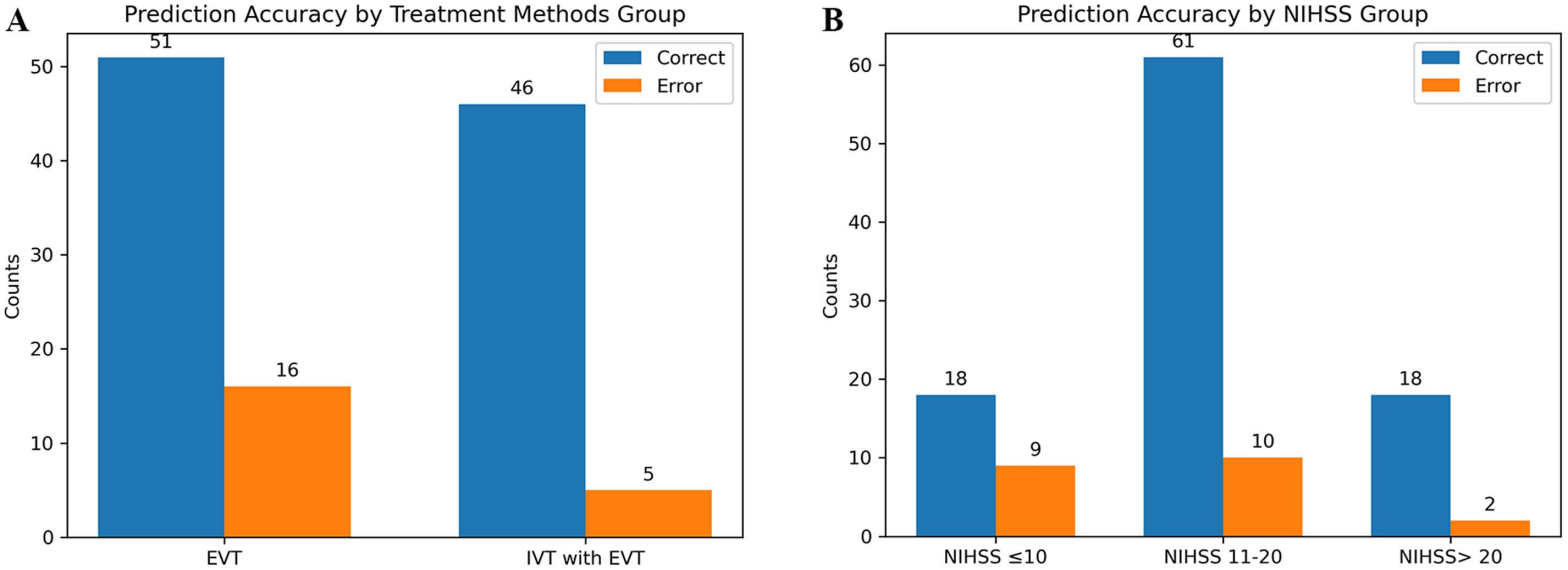
Predictive ability of the combined model in different subgroups. **(A)** Shows the different treatment methods. **(B)** Shows the different stroke severity.

The predictive performance of the combined model in different subgroups was further analyzed and discussed. The model predicted HT with an accuracy of 90.2 and 76.1% in patients receiving EVT with or without thrombolysis, respectively (Figure 6A). The accuracy of the model in predicting HT based on NIHSS scores for patients with different stroke severities was 66.7, 85.9, and 90.0%, respectively (Figure 6B). Additionally, no statistically significant differences were found in model predictions across the different subgroups analyzed (*p* > 0.05).

## Discussion

In this multicenter retrospective cohort study, we explored the correlation between radiomics-based HMCAS on NCCT and HT in AIS patients undergoing EVT, and the results revealed that the radiomics model achieved good predictive efficacy, with an AUC of 0.851 in the test set. Furthermore, we developed a combined model by combining the Rad-score with the radiological risk factor ASPECTS, which further improved the performance of the radiomic model, with an AUC of 0.877 in the test set, and had good calibration and clinical utility.

HT is a common complication and an important prognostic factor after EVT, with an incidence of up to 55.9% (32). In our study, HT occurred in 60.17% of patients, which is higher than previous studies. This may be related to the fact that our study population were patients presenting with HMCAS. Previous studies have shown that a number of clinical factors and imaging features are associated with HT. For example, Hou et al. (33) analyzed patients with large cerebral infarcts without thrombolytic therapy in the HMCAS group and reported that the length of the HMCAS was associated with an increased risk of HT. Yogendrakumar et al. (34) reported that pretreatment ASPECTS, CBS, and clot location were independent risk factors for HT after EVT. Tian et al. (12) included patients from the DIRECT-MT trial who were treated with EVT alone or intravenous thrombolysis combined with EVT and reported that postoperative HT in patients with AIS was associated with a higher glucose level on admission, a longer time from stroke onset to revascularization, the presence of HMCAS, and a lower collateral score. These predictors have not been harmonized and are controversial in clinical studies. Our results revealed that only the imaging feature ASPECTS was an independent predictive marker of HT after EVT, with lower ASPECTS indicating a greater early ischemic extent and a greater risk of HT, similar to the results of previous studies. However, no other variables were associated with HT in this study. The discrepancy may be related to the use of different patient selection criteria and the relatively small sample size.

In recent years, interest in thrombus-based radiomics has increased. Qiu et al. (19) evaluated the value of thrombus radiomics on NCCT and CTA for predicting suitability for intravenous (IV) alteplase recanalization in AIS patients and reported that thrombus heterogeneity was greater in patients who underwent IV alteplase recanalization. Hofmeister et al. (20) showed that first-attempt recanalization with thromboaspiration was associated with a more homogeneous thrombus texture. These two studies reflect the value of thrombus radiomics for recanalization after reperfusion; they focused on analyzing all thrombi, whereas our study was dedicated to HMCAS because it was the most easily identified and outlined on NCCT. Recently, Li et al. (21) extracted RFs of the HMCAS to construct a model, and the results suggested that patients with heterogeneous thrombi had a high likelihood of a poor prognosis. To date, the application of radiomics-based HMCAS for predicting HT after EVT in AIS patients has not been reported. In our work, many RFs were extracted from the HMCAS, and 12 optimal features were identified through screening. The two most relevant features were all from the GLSZM after the wavelet transform (GrayLevelNonUniformity_glszm_wavelet-LLL, LowGrayLevelZoneEmphasis_glszm_wavelet-LHH). LowGrayLevelZoneEmphasis_glszm_wavelet-LHH measures the proportion of low gray level regions among all regions. This feature is negatively correlated with HT, i.e., the larger the value of this feature is, the more low-gray-level regions there are, and the less likely the HT is. GrayLevelNonUniformity_glszm_wavelet-LLL measures the heterogeneity of the gray levels in the image. This feature is positively correlated with HT, which means that thrombi prone to HT after EVT may be more heterogeneous. We speculate that heterogeneous thrombi may be more difficult to remove, leading to more frequent thrombus-removal procedures. Multiple thrombus-removal procedures can cause minor damage to the vascular endothelium and prolong recanalization time, thereby increasing the risk of postoperative HT.

According to the literature, although some studies have explored CT-related radiomics for predicting HT in AIS patients, most of these studies focused on AIS patients treated with thrombolytic therapy or various therapies (15, 35, 36), and only a few studies have investigated the occurrence of HT after EVT. Moreover, the risk of HT is higher in AIS patients undergoing EVT. Recently, Wen et al. (17) developed a CT radiomics model based on selected RFs in the MCA region to predict HT after EVT, with an AUC of 0.797 in the validation cohort. However, our study demonstrated that the classification accuracy of the radiomics model based on the HMCAS alone was good enough (AUC = 0.851) to reliably predict whether patients were in the HT or non-HT group after EVT. Furthermore, the combined model with the addition of imaging features had improved predictive discrimination performance and greater net clinical benefit, although its AUC values were not significantly different from those of the radiomic model. It is reasonable to believe that this may be because RFs are more important than radiological factors and that HMCAS-based RFs have potential for predicting HT. Finally, the model demonstrated good robustness in its predictive performance, including for the analysis of whether or not to combine thrombolytic therapy, as well as for different stroke severity subgroups.

There are several limitations to this study. First, it was a retrospective study, which may be subject to data selection bias. Despite including data from three centers, the small overall sample size and the lack of independent external validation may limit the generalizability of the model. Second, all the ROIs outlined in our study were manually segmented by radiologists, and such an approach is heavily dependent on the radiologist and is time-consuming. Fully automated deep learning-based thrombus segmentation may accelerate clinical decision-making in AIS patients. Third, this study did not categorize HT in detail because of the small sample size. Thus, larger studies are needed. However, the occurrence of cerebral hemorrhage of any type during the postoperative period has an important influence on the clinical outcome. Fourth, because of some incomplete data, we were unable to include some important HT-related predictors, such as the timing of stroke intervention from symptom onset. Fifth, the added value of HMCAS-based radiomics in terms of functional outcome was not analyzed. In the future, we will further expand the sample, collaborate with multiple disciplines, and conduct prospective studies to explore and analyze the clinical value of thrombus radiomics in patients with AIS.

In conclusion, our findings demonstrate the potential of radiomics-based HMCAS on NCCT performed at the time of admission. A combined model that incorporates imaging features could be predictive of post-EVT HT in patients with AIS and facilitate the tailoring of patient-specific interventions by clinicians to optimize stroke management.

## Data Availability

The original contributions presented in the study are included in the article/Supplementary material, further inquiries can be directed to the corresponding authors.
