# Supplementary material for: The value of radiomics-based hyperdense middle cerebral artery sign in predicting hemorrhagic transformation in acute ischemic stroke patients undergoing endovascular treatment
Source: Front Neurol. 2024 Dec 23;15:1492089. doi: 10.3389/fneur.2024.1492089 (PMC11700822; doi:10.3389/fneur.2024.1492089)
Supplement: Supplementary file 1 [file Table_1.DOCX]

Supplementary Material

Radiomics-based hyperdense middle cerebral artery sign predict hemorrhagic transformation in acute ischemic stroke patients undergoing endovascular treatment

Chundan Gong^1,2^, Yun Liu^3^, Wei Ma^2^, Yang Jing^4^, Li Liu^2^, Yan Huang^2^, Jinlin Yang^1^, Chen Feng^5^, Yuan Fang^2*^, Weidong Fang^1*^

*** Correspondence:** Yuan Fang E-mail: nmjyfy@sohu.com
Weidong Fang E-mail: [fwd9707@sina.com](mailto:fwd9707@sina.com)

**Supplementary Table 1.** Summary of CT Scanners and Scanning Parameters for three centers

| Center | equipment | Tube voltage (KV) | Tube current (mA) | Matrix size | Slice thickness  (mm) | Slice  Spacing  (mm) |
| --- | --- | --- | --- | --- | --- | --- |
| 1 | Canon Aquilion one | 120 | 180 | 512×512 | 1 | 1 |
| 2 | GE LightSpeed VCT | 120 | 100-400 | 512×512 | 1.25 | 1.25 |
| 3 | GE New Revolution CT | 120 | 180-235 | 512×512 | 1.25 | 1.25 |

**Supplementary Table 2.** Logistic Regression analysis of independent risk factors for hemorrhagic transformation (HT) in the training cohort.

| Variable | Univariable Analysis | |  | Multivariable Analysis | |
| --- | --- | --- | --- | --- | --- |
|  | OR (95% CI) | P |  | OR (95% CI) | P |
| Sex | 0.576(0.231,1.413) | 0.231 |  |  |  |
| Age | 1.034(0.996,1.076) | 0.089 |  |  |  |
| Stroke etiologies | 0.814(0.352,1.845) | 0.623 |  |  |  |
| CBS | 0.67 (0.496, 0.871) | 0.005 |  |  |  |
| DT | 0.958 (0.917, 0.997) | 0.042 |  |  |  |
| Length of HMCAS | 1.074 (1.02, 1.144) | 0.014 |  |  |  |
| ASPECTS | 0.611 (0.408, 0.837) | 0.007 |  | 0.672 (0.443, 0.946) | 0.039 |
| Drink | 0.542(0.206,1.418) | 0.210 |  |  |  |
| Smoke | 0.875(0.335,2.338) | 0.786 |  |  |  |
| Previous stroke | 1.014(0.231,5.251) | 0.985 |  |  |  |
| Diabetes | 3.798 (1.173, 17.275) | 0.045 |  |  |  |
| Hypertension | 1.339(0.544,3.31) | 0.524 |  |  |  |
| Atrial fibrillation | 0.835(0.295,2.245) | 0.725 |  |  |  |
| Coronary heart disease | 1.714(0.633,5.019) | 0.302 |  |  |  |
| Hyperlipidemia | 2.307(0.514,16.218) | 0.318 |  |  |  |
| History of anticoagulant drugs | 1.646(0.495,6.49) | 0.437 |  |  |  |
| History of antiplatelet drugs | 2(0.542,9.604) | 0.329 |  |  |  |
| Baseline NIHSS score | 1.127 (1.036, 1.241) | 0.009 |  |  |  |
| Glucose level | 1.264 (1.07, 1.576) | 0.017 |  |  |  |
| Triglyceride | 1.149(0.807,2.179) | 0.542 |  |  |  |
| Cholesterol | 0.832(0.571,1.199) | 0.324 |  |  |  |
| HDL | 1.491(0.404,5.872) | 0.554 |  |  |  |
| LDL | 0.646(0.388,1.03) | 0.075 |  |  |  |
| Intravenous thrombolysis | 1.593(0.634,4.166) | 0.329 |  |  |  |
| Thrombectomy modes | 1.409(0.889,2.282) | 0.151 |  |  |  |
| Angioplasty procedure with stent placement | 0.906(0.142,7.187) | 0.917 |  |  |  |
| Number of device＞3 | 0.565(0.144,2.206) | 0.400 |  |  |  |
| Successful recanalization | 0.25(0.013,1.565) | 0.210 |  |  |  |

CBS, clot burden score; DT, distance from the end of the ICA to the thrombus; HMCAS, hyperdense middle cerebral artery sign; ASPECTS, the Alberta Stroke Program Early CT Score; HDL, high-density lipoprotein; LDL, low-density lipoprotein; AUC, area under curve; 95% CI, 95% confidence interval; OR, odds ratio.

**Supplementary Table 3.** Contrasted results of all models in training Cohort and test Cohorts

| Molde Comparation | Training cohort |  | Test cohort |
| --- | --- | --- | --- |
|  | *P* value |  | *P* value |
| Radiological model vs Radiomics model | 0.033 |  | 0.127 |
| Combined model vs Radiomics model | 0.148 |  | 0.424 |
| Radiological model vs Combined model | 0.001 |  | 0.028 |
